# Ulcerogenic risk assessment of diets for pigs in relation to gastric lesion prevalence

**DOI:** 10.1186/1746-6148-9-36

**Published:** 2013-02-22

**Authors:** Maria Grazia Cappai, Maurizio Picciau, Walter Pinna

**Affiliations:** 1 Research Unit of Animal Breeding Sciences of the Agriculture Department, University of Sassari, ex-Faculty of Veterinary Medicine, via Vienna 2, 07100, Sassari, Italy

## Abstract

**Background:**

Gastric ulcers in fattening pigs from intensive pork production can cause sudden deaths on farm and the grinding intensity of the diet appears to be among the risk factors. The objective of this work is to adopt the latest laboratory tests and thresholds for the ulcerogenic risk assessment of diets from experimental reports and verify the class of risk in relation to gastric lesion prevalence in reared finishers.

**Results:**

Specificity and accuracy of feed safety tests based on the ulcerogenic risk of feed associated with the particle size distribution of diets were calculated on the occurrence of gastric lesions observed at a slaughterhouse: 41 lard-type hogs, fed with two diets [pelleted (n = 21 pigs) *vs*. mixed meal (n = 20 pigs)], analyzed at the laboratory of our Institute, were involved. Gross inspection at the abattoir allowed the identification of the development of macroscopic gastric lesions in the pigs (13/21) fed with a pelleted complete diet, ranked in Class 1 (high ulcerogenic risk) on laboratory assessment. Breakdown of gastric lesion severity: hyperkeratosis (13/13), mucosal erosions (11/13) and bleeding ulcers (2/13). This occurrence was compared to the morphology of stomach mucosa from 20 finishers fed with a mixed meal diet, ranked in Class 3 (low ulcerogenic risk), in which no gastric lesions were observed. Very fine particle (VFP) mass (<0.4 mm) according to cut off thresholds (>36%) for the safety ranking of diets, showed: 100% positive predictive value (PPV); 100% specificity; 88.1% accuracy; 72.2% sensitivity.

**Conclusions:**

Three factors emerged: the elevated mass (42.6%) of <0.4 mm particles in the pelleted complete diet confirmed the associated risk rank in Class 1 assessed by laboratory procedures, as gastric lesions were selectively observed in 61.9% of finishers fed with the high risk diet; in these animals, macroscopic gastric lesions occurred within four weeks and showed a sub-clinical course, independently of severity; proper sieving analysis is necessary to define the VFP proportion in feedstuffs with certainty, as an adequate measure to assess the ulcerogenic risk class of the diet.

## Background

Lard-type hog production is widespread in the Mediterranean region [1-6]: this semi-extensive husbandry is mostly concentrated in a few farms, with a production of between 20–40 hogs per week, slaughtered at a final body weight of between 125–135 kg [7]. Finishers are commonly fed on mixed meal diets based on raw feedstuffs, often soaked, composed of shredded cereals, legume seeds – meal, acorns and, not rarely, on grassland. Nevertheless, in recent years, the use of manufactured complete feed for finishing pigs is becoming ever more popular: some farmers are compelled to range finishers and change the feeding practice to comply with strict regulations aiming to limit and control the spread of African Swine Fever infections. Therefore, homegrown feedstuffs are collected and administered to pigs, but sometimes farmers turn to manufactured feedstuffs, to ease handling, storage and increase feed conversion efficiency.

The amount of pelleted feed sent to the service section of our institute for quality assessment has recently increased in a proportional manner. Among the parameters screened for during quality assessment and the composition of samples, grinding intensity is routinely checked in pelleted feed, as it is an issue in animal welfare [8] and public health [9,10] concerns.

As a matter of fact, gastric lesions are spreading in intensive pork production with a wide range of prevalence (32%–65%; [11]), mostly affecting the non glandular gastric (NGR) mucosa (fairly extended in pigs’ stomachs) in slaughtered hogs. Epidemiological data about “in farm” sudden deaths (1–2%) from bleeding gastric ulcers are reported for pigs mainly from three to six months of age. The cause of gastric ulceration is not clearly understood, but the grinding intensity of the diet appears to be within the list of risk factors [11-13]. Recently, advances in the etiology and pathogenesis of pigs’ gastric lesions have supplied significant tools to support veterinary practitioners in the evaluation of risks associated with the diet. These can be listed as follows: 1) the practice of increasing the proportion of coarse particles in the diet gives no ulceroprotection when the very fine particle (VFP) proportion is also greatly represented [8] and, especially in pelleted feed, coarser particles contribute to making the pellet prone to instability [14]; 2) the particle size distribution should be examined after the pelleting process as it represents a second milling [14]: as a matter of fact, the grinding phase of the manufacturing process, before pelleting, concurs to the particle size of the pellet, but the pelleting phase finalize it. Thus, the pigs could ingest higher proportions of fine particles with the pelleted diets than set in the grinding phase of the production line; 3) the VFP (<0.4 mm) proportion has been identified as a “decisive factor” and that experimental evidence suggests we should focus on this proportion of particle mass in relation to the potential ulcerogenic role exerted by the diet [8-10,15].

In the light of such developments, a considerable achievement in laboratory procedures is that of the recent clarification of the sieving method, in relation to feed samples’ physical form: the particle size distribution obtained by sieve analysis (dry/wet) shows statistical significance (P < 0.05 on determination of the% of particles <0.4 mm; [14]) between results if dry *vs*. wet sieve analyses are used to process meal *vs*. pelleted diets, respectively.

On farm effects from a pelleted complete diet, ranked Class 1 (high ulcerogenic risk) *vs.* a mixed meal diet, ranked Class 3 (low ulcerogenic risk), for finishing pigs from the same farm for lard-type hog production, were investigated and correlated to gastric lesions found at the slaughterhouse: the specificity and accuracy of laboratory tests for “abiotic feed safety” assessment based on the feed’s ulcerogenic risk associated with the particle size distribution of the diet in relation to morphological changes in the stomach’s mucosa were determined.

## Results

### Quality of feedstuffs

**Figure 1:**
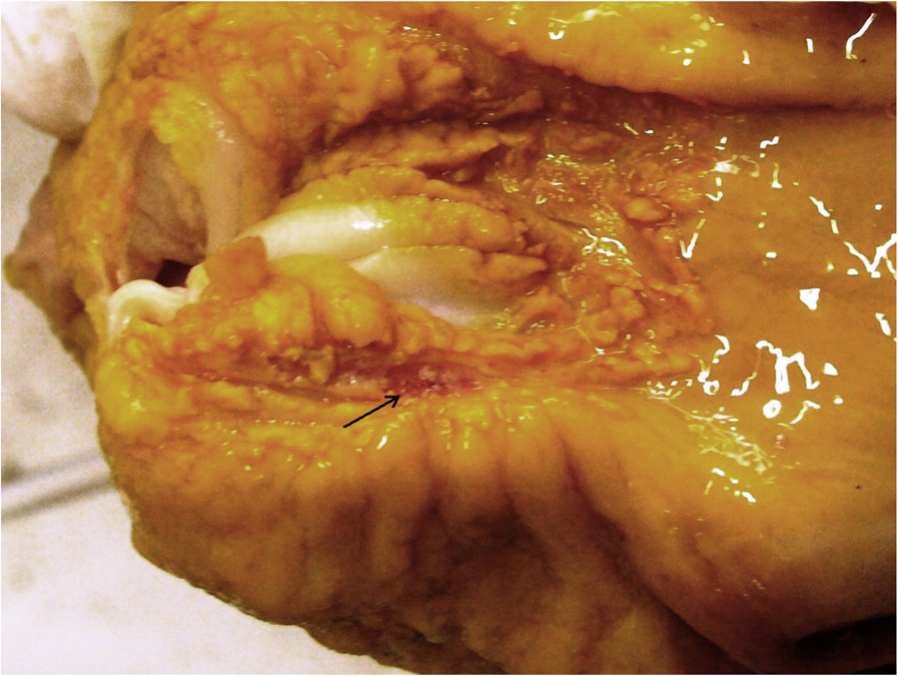
Bleeding gastric ulcers and gastric erosions between esophageal mucosa crests showing hyperkeratosis, in a pig from the pellet fed group.

**Figure 2:**
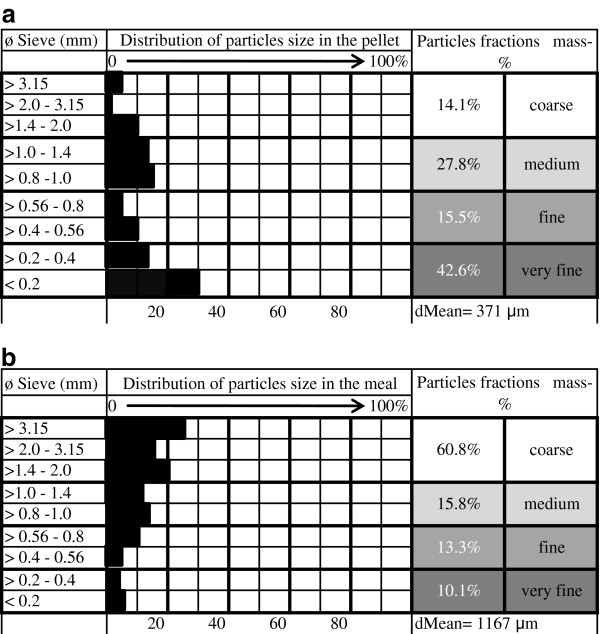
Particle size distribution of the pelleted (a) and the mixed meal (b) diets, for finishing pigs.

The pelleted and mixed meal diets were of satisfactory quality and proper hygienic status. Cylindrical pellets of 3.5 mm in size and a 42.6% of VFP (<0.4 mm) mass characterized the structure (Figure 2a) and set the pellet in the risk Class 1. The mixed meal diet ranked in risk Class 3: the analysis performed on the mixed meal diet highlighted a very low amount of VFP (10.1% < 0.4 mm), considerably below the lower cut off value associated with ulcerogenic risk from the diet reported in the literature (Figure 1b). A condensed value of the averaged granulometry of the diets is reported in Figure 1 as a geometric mean. The chemical composition of the two diets is reported in Table 1.

**Table 1:** Chemical composition of diets

**Chemical diet composition**	**Mixed meal diet**	**Pelleted diet**
Finishers	20	21
Dry matter (g/kg, as fed)	882	890
Ash (g/kg DM)	60.9	68.4
Crude protein (g/kg DM)	144	175
Crude fibre (g/kg DM)	43.2	45.0
Crude fat (g/kg DM)	29.8	22.8
Starch (g/kg DM)	424	435
OM (g/kg DM)	939	931

### Animal performance

During clinical inspections both on the farm and in the abattoir before slaughter, all of the animals appeared healthy. The totality of pigs started to enter the slaughtering chain one by one, after one hour and 12 minutes from their arrival at the abattoir. The speed of the slaughtering chain ranged between four to six minutes per pig: such variation depended on the time needed for the phases of stunning and bleeding at the beginning of the line. All the pigs got slaughtered within the sixth hour from being held off feeding. The following productive performance were determined and calculated: body weight (BW: 129 ~ 135 kg) at slaughter; carcass yield was 77.6% – 78.8%; back fat thickness in the maximum width was 3.9 – 4.2 cm, showing good fatness of the carcass (see Table 2) in all the animals: however, it is to be pointed out that the animals fed with the pelleted diet showed a more favorable feed conversion ratio (FCR) and better yields, although the differences were not found to be statistically significant (Table 2). Thus, the economic advantage constituted by the improved feed conversion efficiency of the pelleted diet is clearly a factor which breeders will want to take into account.

**Table 2:** Animal performance in pigs fed with the different diets during the trial

**Diet**	**Mixed meal**	**Pelleted diet**
**Finishers**	**20**	**21**
**Animal performance**		
Body weight at start (kg)	113 ± 3.47	113 ± 4.12
Final body weight (kg)	131 ± 2.85	137 ± 1.89
Feed consumption, *g*^*as fed* × *d*–1^	3219 ± 199	2997 ± 340
BW gain, % *of BW at start* × 28*d*^–1^	15.9	21.2
FCR, *g* × *g*^–1^	5	3.34
Carcass weight (kg)	103 ± 0.89	107 ± 0.60
Carcass yield (%)	78.6	78.1
Back fat maximum width (mm range)	4.35 ± 0.45	4.20 ± 0.20

### Anatomo-pathological findings

Macroscopic lesions were found in 13 out of the 21 pigs fed with the risk Class 1 diet. These were observed in the NGR mucosa and were classified as hyperkeratosis (13/13), mucosal erosions (11/13) and bleeding ulcers (2/13). No pathological signs were macroscopically detected in the remaining areas of gastric mucosa. Following detailed examination, multiple erosions appeared to fuse together into more and more extended areas, resulting into bleeding gastric ulcers, typically found at the edges of the NGR mucosa, by the GGR mucosa (Figure 1, see arrow). This site is typical for gastric ulcers as a pathognomonic sign of a physico-chemical injury in the stomach of the pig: acidity of the chyme and impaired/reduced buffering activity of the secretory mucosa to preserve NGR from tissue damage, leading to similar gross morphological changes. A dietary origin in the development of such injuries can be reasonably supposed and may provoke a predisposition to secondary colonization by infectious agents (*Helicobacter* spp.) in the NGR mucosa. Nevertheless, a different tropism in different areas of the stomach (glandular gastric region, GGM or pyloric gastric region, PGR) was assessed in agreement with different bacterial ulcerogenic strains in the pig. Direct ulcerogenicity of the diet cannot be attributed with certainty, as no linear effects were estimated in this experience; furthermore, the diets differed not only in terms of particle size distribution, but also in the composition of ingredients and physical form: nevertheless, the quality assessment of diets did not highlight any particular dietary risk, neither were there any particular ingredient peculiarities or their combination which could be linked to increased ulcerogenic risk for the pig on the basis of reports in the literature, apart from the intensely ground pelleted diet.

The differential diagnosis took into consideration the search for *Hyostrongylus rubidus* worms*,* detectable in stomach mucosa at gross inspection, with negative response in all pigs from both groups.

No macroscopic pathological signs were detected in stomachs from pigs (20/20) fed with the mixed meal diet.

The microscopic evaluation of NGR mucosa in apparently healthy stomachs from both groups of animals, allowed us to reveal true negative samples in the totality of pigs fed with the meal diet, while 5 out of 9 samples were positive responses for tissue damage and were considered false negative samples (because undetected on gross inspection of the stomachs at the slaughterhouse) with regards to pigs fed with the pelleted diet. At light microscopy, inflammatory cells stiffen the gastric mucosa from the *stratum corneum* throughout the *lamina propria*, in agreement with the grade of severity. The presence of lymphocytes, plasma cells and neutrophil granulocytes were observed: the chronic lesions (abundance of lymphocytes) observed in the epithelium of the NGR mucosa showed also reiterated and continued injury (neutrophils presence), up to bleeding ulcers.

### Class of risk tests

According to the prevalence of gastric lesions observed and the cut off threshold reported in the literature, the methodology for Class of risk assessment based on the VFP proportion from both meal and pelleted diets turned out to have a strong positive predictive value (PPV: 100%) and specificity or true negative rate (TNR: 100%) followed by good accuracy (ACC: 88.1%) and sensitivity or true positive rate (TPR: 72.2%). The supply for 4 weeks of the finely ground pelleted diet showing a high proportion (42.6%) of VFPs (far beyond the value of 36%, reported in the literature as being the threshold beyond which a predisposition to macroscopic gastric lesions may develop) was associated with sub-clinical and non-impacting on productive performance macroscopic gastric lesions in 61.9% of the specimens. This is in agreement with the high ulcerogenic risk (Class 1) assessed for the pelleted diet on laboratory estimation of the risk.

## Discussion

Grinding intensity negatively impacts on the stomach’s mucosal integrity and prophylactic and therapeutic recommendations suggest a lower grinding intensity of the diet [11,16]. The potential pathogenesis described in the literature was linked to a more fluid stomach content and the consequent fall of the pH gradient between the proximal and the distal parts of the stomach: the anatomical structure of the proximal part of the stomach (NGR) in the pig represents a weak point. Predominantly, tissue damage takes place as no mucous protection is assured, due to the lack of secretory cells in the *esophageal* mucosa, prone to developing bleeding gastric ulcers more easily. Moreover, strong grinding intensity of the pellet and heating treatments to increase nutrient digestibility rates (in particular of starch), determine the gelatinization of starch and a consequent increased viscosity of the gastric content, favoring the contact between the acid chyme and the NGR mucosa. In this experience, though bleeding gastric ulcers were found in slaughtered pigs fed with the pelleted diet, it is presumable that in the near future they could have acted as the cause for sudden deaths on farm: as reported in the literature, pigs with extensive and severe erosive lesions of the *pars oesophagea* showed to be healthy at the clinical examination [17], in case of minimal blood losses. At this regard, during the clinical examinations carried out on the totality of pigs before slaughter, neither pale pigs nor symptoms attributable to blood losses could be assessed. In addition, *in vivo* performance of pigs fed with the pelleted diet turned out to be more favourable than those of pigs fed with the mixed meal diet, in terms of body weight at slaughter and feed conversion ratio. On the other hand, Friendship [17] reported also that the blood loss could be severe and acute: thus, in some cases, pigs can be found dead before any clinical sign could be detected. The bleeding ulcers found in two out of 21 pigs fed with the pelleted diet displayed a mild blood loss: in addition, no large coagula were found in the stomach contents, but trails of blood. The finding of the bleeding ulcers was attributed to the progressive coalescence of close mucosal erosions, advancing between the tissue with hyperkeratosis, typically at the edge of the *pars oesophagea*.

In addition, the dietary role in the pathogenesis of gastric ulceration in pigs should take into account various factors: ingredients (botanical origin of cereals used as ingredients, because different milling properties are linked to genetic types of kernels); heat/pressure treatment (starch gelatinization); grinding intensity (above all whether associated with heating treatment or not, due to starch viscosity changes), and considering that the pelleting process represents a second milling effect itself. Due to such a multi-factorial approach to define the relationship between the dietary role and the development of gastric ulcers in pigs, the most reliable test should consider: a) the cereal composition; b) the pre-pelleting grinding intensity of feedstuffs and c) the pelleting process itself, as co-factors predisposing to the onset of injuries in the NGR of the stomach of the pig. As a consequence, a first discrimination on the potential ulcerogenic risk of the diet should focus on the manufacturing process: the grinding intensity in the pre-pelleting phase is a crucial point in the resultant particle size distribution of the diet (further micromilling as an effect from the pelleting process). Therefore, the quality of the physical form of the diet should be assessed both on the grinding intensity (coarse to fine) and on the compaction (meal *vs*. pellet). Nevertheless, it is to be underlined the fact that commonly the particle size distribution is assessed on feedstuffs from the market, as final products, when the pelleting phase already occurred: an appropriate management of the ulcerogenic risk of diet should address the grinding intensity in the pre-pelleting phase of the process as a critical control point (CCP). According to the latest procedures described in the literature, the laboratory method to assess the granulometry of the feed should adopt a dry *vs.* wet sieve analyses when processing meal *vs.* pelleted diets, respectively [14]: an inappropriate sieving method would lead to statistically significant differences in the determination of micromilled particles (<0.4 mm) mass in feedstuffs with different physical form. The deployment of dry, rather than wet, sieve analysis in a pelleted diet leads to a bias towards the underestimation of the proportion of VFPs in the range of from one third to one half, determined on the same pellet sample [14] with wet sieving.

Moreover, an average value to express the particle size of the feed appears to be no longer informative about the possible risk associated with the diet, in the light of the latest results from experimental trials and from this field experience: increased proportions of coarse particles showed no prophylactic effect in the ulceroprotection, when high proportions of VFPs also characterized the same pelleted diet. Therefore, an “overall” value to express the granulometry of feedstuffs could conceal the real ulcerogenic risk associated with the diet, if the proportion of VFPs is not stated. The literature reports a cut off level concerning the increased prevalence of gastric ulcers when the feed is of a granulometry smaller than 700 μm [11]. To what extent the prevalence of gastric lesions increases with particle size smaller than 700 μm appears to be unstated to date. Moreover, if greater proportions of coarse particles are repre1sented, the average value changes but no ulceroprotection is given. This means that the average value is not as specific and accurate as the VFP proportion to correlate with the ulcerogenic risk of the diet, potentially involved in gastric ulcer development in the pig.

Finally, infectious agents leading to gastric ulceration were not given primary consideration because the dietary role appeared to be involved in the failure of the stomach’s mucosal barrier and self-protecting/healing capability: the literature reports that helicobacter-like strains are widespread populations in pigs’ stomachs. Nevertheless, some strains appear more pathogenic than others as their presence has also been linked to gastric ulceration [17-20], but only occasionally and in different gastric regions from NGR mucosa. In any case, due to the healthiness of finishers fed with the meal diet showing a constant integrity of NGR mucosa, the infectious agents were not investigated further.

According to the high rate of the predictive positive value, which reflects the precision of the test, the determination of VFP proportion of the diet was used as a screen test to assess the high risk of developing gastric lesions, due to null false positive results (gastric lesions from the meal diet, far below the cut off value, were 0 out of 20). Moreover, the high specificity of the test correctly identified that low VFP values in the diet caused no gastric lesions. As far as sensitivity is concerned, the correctly predicted association with 72.2% of gastric lesions is probably a time dependent parameter: in fact, false negatives at gross inspection were revealed only after microscopic investigation. With regards to the exposure to the risk Class 1 diet, it is to be highlighted that the switch to coarsely ground diets is reported to improve stomach conditions and facilitate the healing process [13]. Therefore, the adoption of the laboratory procedures for the quality assessment of feedstuffs aiming at determining the particle size distribution of a new diet for pigs, might be a reliable preventive measure to reduce the risk of gastric lesion onset in the intensive pork production. In relation to the accuracy and precision rates obtained according to the prevalence of gastric lesions observed to assess the feed ulcerogenic risk, the determination of the VFP proportion in the diet can be considered a valid test.

## Conclusions

The cut off limit reported in the literature concerning VFP proportions in the diet associated with high risk of the development of gastric lesions, appeared to be reliable in this field experience: such a proportion might be a valuable indicator to estimate the potential risk gastric lesion onset in the pig, related to the the diet. Nevertheless, the most appropriate management of the ulcerogenic risk from pelleted diets resides in lowering the grinding intensity of the raw ingredients, before pelleting; on the other hand, a good compromise should be achieved during the grinding phase of the manufacturing process, not to affect the stability of the pellet. The method based on the wet sieve analysis to assess the particle size distribution of the pelleted diet, sampled in the swine farm, appeared to be useful in practice and reliable for the determination of the VFP proportion of pelleted diets. As a practical concern in swine husbandry, preventive measures should take into account the fact that bleeding ulcers in the NGR mucosa of pigs’ stomachs can be clinical endpoints of a basic progressive pathologic status, showing a sub-clinical course. It is suggested that routine analyses of feedstuffs should include particle size distribution assessment: the assessment of the class of risk predisposing to gastric lesion development based on the VFP proportion of the diet might be useful to prevent gastric ulcers, sudden deaths and economic losses at herd level.

## Methods

### Animals and diets

Animal handling followed the recommendations of European Union directive 86/609/EEC and Italian law 116/92 concerning animal care. The study was approved the by the Ethical Committee of the University of Sassari for Research on Experimental Animals (Approval no. 20429/X/10/5, 2012).

The study involved a total of 41 crossbred (Duroc sires on Italian Large White X German Landrace) finishers, individually identified, from the same farm with a body weight at the start of the finishing period between 107 and 119 kg. The animals were reared indoors, at a temperature of from 21 and 24°C and relative humidity of 61.5% to 67%, and housed in groups of 5–6 pigs per squared pen (6 × 4 mt.) on grates. Finishers were fed two different diets: 20 pigs were fed a conventional mixed meal diet, based on ground barley and pea meal, according to the farmer's recipe; 21 hogs were fed a pelleted complete feed for finishing pigs, deployed for the first time on the farm. The feeding plan lasted four weeks, until all the 41 pigs were slaughtered in the same abattoir. All the pigs were moved from the pens and transported to the abattoir, 60 km far from the farm: both groups were held off feeding throughout the duration of the transportation and during the staying in the lairage at abattoir, before entering the slaughtering chain.

In the last two weeks, averaged daily feed intake was reported per pen and calculated per pig grouped in the same pen, for feed conversion efficiency estimation.

### Laboratory procedures and quality assessment of feedstuffs

#### Quality assessment of feedstuffs

The farmer sent a sample of the pelleted diet for finishers to the service section of our institute for quality assessment. During laboratory testing, the feed sample underwent sensory analysis based on the outer evaluation: colour, odour and shape, correlated to stability. The stability was assessed on the pellet’s dusting, powdering or falling into pieces: by handling the pellet, constant shape based on powder transfer to the operator’s hands, was limited to intense powdering/crumbling-scored. The hygienic status assessment was based on recommendations for shred and pelleted feedstuffs [21]: the potential presence of parasites (mites and other insects, either adults or larvae) and moulds was investigated. The diameter size of the pellets was measured by digital callipers. The particle size distribution was assessed by dry *vs*. wet sieve analysis [9,10], according to the latest procedures described by Wolf et al. [14] when shred or pelleted feedstuffs have to be characterized. The wet sieve analysis required 50 g of pelleted sample. The wet sieve analysis and the particle size distribution was performed by means of an 8 sieve-tower (mesh size, mm: 3.15, 1.4, 1.0, 0.8, 0.6, 0.4, 0.2). The sample was put for 1 h in 1000 ml of water (30°C, agitation after 30 min) and the feed-water-suspension was cast on the top sieve of a tower which was placed on an open topped vessel with a run-out. This sample was rinsed with 10 l of cold, distilled water (pressure: 1 bar). The sieve tower was dried in a cabinet dryer (103°C), until it reached a constant weight.

#### Chemical composition of diets

An amount of 150 g of both samples was oven-dried (103°C) and ground (0.5 mm): the samples were analyzed in duplicate and chemical composition determined by modified Weende analysis [22]. The crude protein (CP) content was assessed by the Kieldahl method.

### Animal performance, health status and post mortem inspection

#### Animal performance and health status

At the end of the finishing period, after four weeks of feeding with the pelleted diet and the mixed meal diet, all the pigs were monitored on the farm, before slaughter: during the clinical examination, health conditions were compared between the finishers fed with the different diets. Both groups of animals had also been monitored for daily body weight gain (gd^-1^), feed conversion ratio and final body weight at slaughter (kg). Animals entered the slaughtering chain without a particular order, as, progressively, the Veterinary Service of the abattoir completed the check list of provenience and the clinical examination of animals, in agreement with the Italian D.P.R. 317/96, IV mod., modified by D.M. 16^th^ of May, 2006 for the accomplishment of the European Directive 92/102/CEE.

At the end of the slaughtering chain, carcass weight and warm carcass yield were calculated and back-fat thickness measured by means of a calliper in the maximum width (between the third and fourth rib, six cm from the spine).

#### *Post mortem* inspection

At the end of the slaughtering chain, the usual *post mortem* inspection of the carcass and viscera was performed on all specimens. The gastric mucosa from each carcass was inspected directly at the abattoir, to identify the potential presence of gastric lesions in both groups of animals fed the pelleted *vs*. meal diets. The stomachs were promptly removed from the carcass and excised from the oesophagus and the duodenum, after double ties. Separately, stomachs were opened at the big curvature and each content collected. Internal mucosa was exteriorized and rinsed in cold water to remove remaining ingesta and permit gross inspection.

#### Macroscopic evaluation of stomach mucosa

**Figure 3:**
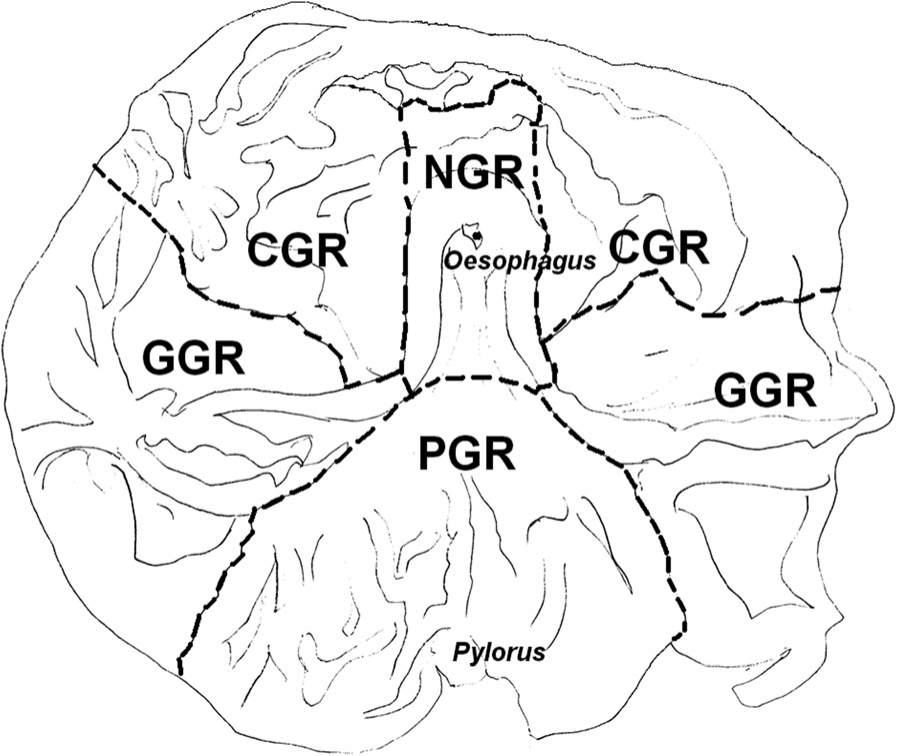
Regions of the gastric mucosa of the pig, by Maria Grazia Cappai 2011.

A first parameter considered the regions of the stomach, named in a progressive oral to aboral-order: non glandular region (NGR), also called oesophageal mucosa, considerably large in the stomach of the pig; cardiac glandular region (CGR), gastric glandular region (GGR) and pyloric glandular region (PGR), as reported in the drawing from the original internal stomach wall of the pig (Figure 3, by M.G. Cappai, 2011). The identification of the mucosal regions is necessary to describe the spot where gastric lesions might occur. Lesions were classified according to morphological changes in the gastric mucosa: hyperkeratosis, erosions or ulcers. Moreover, inflammatory processes were also considered.

#### Microscopic examination

Samples from the apparently healthy non glandular regions (NGR) of stomach mucosa from all the pigs were processed for microscopic examination. Tissue cuts were immediately washed and fixed in formaldehyde (10%) for histological analysis. After dehydration and paraffin embedding of the tissues, 2 μm thick sections were stained using Haematoxylin-Eosin (HE) and Periodic Acid’s Schiff stain (PAS). Slides were examined by optical microscopy (Leica CME 5221).

### Calculations and statistical analysis

During the laboratory assessment of particle size distribution of diets, the calculation of each sieve fraction was based on the dry matter content of the diet and expressed as mass proportion. Particles were calculated and added to the fraction of particles below 0.4 mm of diemeter. According to the different mesh sizes of the eight-sieved tower, four qualitative ranks were identified: coarse particles (% ≥1.4 mm); medium particles (0.8 mm ≤% <1.4 mm); fine particles (0.4 mm ≤% <0.8 mm); and very fine particles VFP (% <0.4 mm). Cut off levels of VFP mass of diets were assessed [8]: over 29% of particles <0.4 mm, microscopic mucosal lesions; over 36% particles < 0.4 mm, macroscopic gastric lesions. On this basis, three classes of ulcerogenic risk of diets were therefore assessment:

Class 1, high risk (VFP > 36%);

Class 2, moderate risk (29% < VFP <36%);

Class 3, low risk (VFP <29%).

The confidence of cut off levels of VFPs in diets was related to the prevalence (P, %) of the gastric lesions encountered, calculated in each group of animals as diet-dependent:

$$\mathrm{Prevalence}:P=\frac{n_{s}^{l}}{\left(n_{s}^{l}+n_{s}^{h}\right)}\times 100,$$

where *n*_*s*_^*l*^ = number of stomachs with lesions, *n*_*h*_^*s*^ = number of apparently healthy stomachs.

Furthermore, positive predictive value (PPV), sensitivity (or true positive rate, TPR), specificity (or true negative rate, TNR) and accuracy (ACC) of the very fine particle mass determined on diets through wet sieve analyses based on cut-off levels were calculated using the following formulas:

$$\mathrm{PPV}=\frac{\mathrm{TP}}{\left(\mathrm{TP}+\mathrm{FP}\right)}\times 100;$$

$$\mathrm{TPR}=\frac{\mathrm{TP}}{\left(\mathrm{FP}+\mathrm{TN}\right)}\times 100;$$

$$\mathrm{TNR}=\frac{\mathrm{TN}}{\left(\mathrm{FP}+\mathrm{TN}\right)}\times 100;$$

$$\mathrm{Acc}=\frac{\left(\mathrm{TP}+\mathrm{TN}\right)}{\left(\mathrm{TP}+\mathrm{TN}+\mathrm{FP}+\mathrm{FN}\right)}\times 100,$$

where TP is the number of stomachs with macroscopic gastric lesions from pigs fed with the pelleted diet, FP is the number of stomachs with macroscopic gastric lesions from pigs fed with the mixed meal diet, TN is the number of stomachs with no lesions in all the pigs, FN is the number of stomachs that were apparently healthy but with microscopic tissue damage.

As far as animal performance is concerned, statistic significance between groups was assessed using Student’s T-test.

## Competing interests

The authors declare that they have no conflict of interests.

## Authors’ contributions

MGC participated in the design of the study and performed feedstuff evaluation, histological investigations, calculations and statistical analysis. MP participated in fieldwork activities both on the farm and at the abattoir. WP conceived of the study, and participated in its design and coordination and helped to draft the manuscript. All authors read and approved the final manuscript.
